# The Effectiveness of COVID-19 Vaccines in Preventing Hospitalizations During the Delta Wave: A Patient-Population Study at a Major Referral Center

**DOI:** 10.7759/cureus.26030

**Published:** 2022-06-17

**Authors:** Ahmad Salman, Ghaidaa Elsaddik, Zeinab El Mawla, Rim Masri, Matina Hamadeh, Amena Khatoon, Michelle W Saliba, Afaf Michel Minari, Mahmoud Hassoun, Pierre Abi Hanna

**Affiliations:** 1 Infectious Diseases, Lebanese University Faculty of Medicine, Beirut, LBN; 2 Internal Medicine, Beirut Arabic University, Beirut, LBN; 3 Internal Medicine, Lebanese University Faculty of Medicine, Beirut, LBN; 4 Infectious Diseases, Rafik Hariri University Hospital, Beirut, LBN; 5 Pulmonary and Critical Care Medicine, Rafik Hariri University Hospital, Beirut, LBN

**Keywords:** hospitalization, lebanon, immunization, vaccination, covid-19

## Abstract

Background and objective

Coronavirus disease 2019 (COVID-19) has turned into a deadly global pandemic since its first discovery in Wuhan, China in December 2019. Safe and effective vaccines against COVID-19 have been introduced to the public and have been shown to reduce the severity of the disease and related mortality rates. COVID-19 vaccination was first introduced in Lebanon in mid-February 2021. In this study, we analyzed the effectiveness of vaccination against COVID-19-related hospitalization during the Delta wave at a major referral center in Lebanon.

Methods

This patient-population study was conducted on patients hospitalized with COVID-19 between July 1, 2021, and September 30, 2021, at the Rafik Hariri University Hospital (RHUH) in Beirut, Lebanon. Data were collected directly from the patients or from digitized records and included demographic characteristics (age, sex, and comorbidities), vaccination status, oxygen requirement, and outcomes. National vaccination data were collected from the daily bulletin provided by the Lebanese Ministry of Public Health. The data collected were analyzed using SPSS Statistics Version 19.0 (IBM Corp., Armonk, NY).

Results

A total of 289 patients were included in the study, of whom 90.3% were unvaccinated and 9.7% were vaccinated with at least one dose of a two-dose regimen. Only 4.5% of the 289 patients were fully vaccinated. Among those fully or partially vaccinated, the mean time from symptom onset to hospitalization was shorter but the hospital stay was longer compared to the unvaccinated group. The mortality rate was higher in the unvaccinated group (25.7%) compared to 14.3% among the vaccinated. The vaccine effectiveness compared to the national vaccination rate (22.5% in the population after the first and second dose) was 71.71% and 83.78% respectively.

Conclusion

The findings of this study highlight the fact that complete/partial vaccination against COVID-19 was highly protective against severe disease and hospitalization during the period with a predominance of the Delta variant in Lebanon.

## Introduction

Coronavirus disease 2019 (COVID-19), caused by the novel severe acute respiratory syndrome coronavirus 2 (SARS-CoV-2), has led to one of the most lethal pandemics in history. The virus itself was first isolated in December 2019 in Wuhan, China. Ever since then, the number of reported infected cases has reached more than 520 million as of March 13, 2022, with a fatality rate of more than six million. The disease has been reported in 213 countries/territories on a daily basis [1].

The clinical presentation of COVID-19 is highly variable, ranging from asymptomatic infection to severe forms of respiratory failure and multi-organ involvement. Efforts to understand the COVID-19 pathophysiology and global clinical trials have led to major advancements in the medical management of the disease and resulted in favorable outcomes for a considerable segment of the patients [2].

Despite many non-pharmacological interventions, such as surveillance, travel bans, avoiding mass gatherings, lockdowns, and using face masks, the pandemic has spread globally in a rapid manner and has had a tremendous impact on people’s health and well-being [3]. This unprecedented spread accelerated the demand for devising vaccines that could safely and effectively prevent the disease or reduce its progression to a more severe state. By January 2022, WHO had issued emergency use authorizations for nine vaccines against COVID-19, including the Pfizer/BioNTech vaccine and the Oxford AstraZeneca vaccine among others [4]. Several clinical trials have established that the approved vaccines appear to be safe and are critically effective tools in preventing severe forms of COVID-19, hospitalizations, and mortality related to all variants of the disease so far [5]. The efficacy/effectiveness against the symptomatic disease has been heterogeneous between different vaccines and studies but has almost always been greater than 50% and often higher than 90% [3].

As of March 9, 2022, 1,081,459 COVID-19 infections and 10,176 deaths have been reported in Lebanon since the pandemic started. More than five million doses of COVID-19 vaccines (including first, second, and third doses of multi-dose regimens) have been administered so far [6]. In Lebanon, vaccination against COVID-19 started in mid-February 2021. The country has relied mainly on the Pfizer/BioNTech BNT162b2 mRNA vaccine followed by the Oxford AstraZeneca ChAdOx1 nCoV-19 adenovirus vector vaccine (AZD1222). A very small percentage of the vaccinated population received Russian or Chinese vaccines. In this study, we employed the patient-population method to analyze the effectiveness of vaccination against COVID-19-related hospitalizations [6].

## Materials and methods

Study design 

This was a retrospective study about the effectiveness of COVID-19 immunization conducted among hospitalized patients between July 1, 2021, and September 30, 2021, at the Rafik Hariri University Hospital (RHUH) in Beirut, the largest public hospital in Lebanon and a major referral center for COVID-19.

Study population

Definitions

Fully vaccinated: patients with onset of symptoms at least two weeks after receiving the second dose of vaccine.

Partially vaccinated: patients who have received only one dose of vaccine before the diagnosis or those with the onset of symptoms less than 14 days after the second dose.

Unvaccinated: patients who have not received any dose of vaccine.

Sample Size

The sample size was 289 patients, including all eligible subjects.

Regarding the selection of the study subjects, all consecutive patients aged ≥18 years hospitalized for COVID-19-related hypoxemia or other related severe symptoms during the study period were included in the study. Also, the inpatients who acquired severe COVID-19 during the study period were also included. The diagnosis of the cases was confirmed by a SARS-CoV-2 RT-PCR test.

Data collection 

Medical charts were reviewed by employing a consistent dataset to collect data related to demographic characteristics (age, sex, and comorbidities), vaccination status, oxygen requirement, and outcomes. The basic data were collected from the charts. In case of any missing data, the participants were contacted by phone (on their numbers collected from the charts) to complete the study part concerning the vaccination status.

The study received ethical approval from the Institutional Review Board (IRB) approval from the IRB at RHUH. All the information collected has been fully anonymized to ensure patient confidentiality.

Vaccine effectiveness was calculated by using the following formula [7]:

Vaccine effectiveness = (incidence in unvaccinated population - incidence in vaccinated population) x 100% / incidence in unvaccinated population.

Statistical analysis

The data collected were analyzed using the SPSS Statistics software Version 19.0 (IBM Corp., Armonk, NY) and were described using frequencies (N), percentages (%), means, and standard deviations (SD). Absolute numbers and percentages were used to report categorical variables. Chi-square distribution was employed to determine the association between categorical variables. All data were subjected to two-sided analysis and a p-value ≤0.05 was considered statistically significant.

## Results

A total of 289 patients were included in the study with a mean age of 53.88 years (range: 16-98 years); 59.5% of the patients were female. The nationalities included were mainly Lebanese (67.5%) and Syrian (23.9%), while the remaining included Palestinians, Iraqis, and other nationalities. The data relating to the length of stay were as follows: mean: 12 days, median: eight days, and range: 1-195 days.

**Figure 1:**
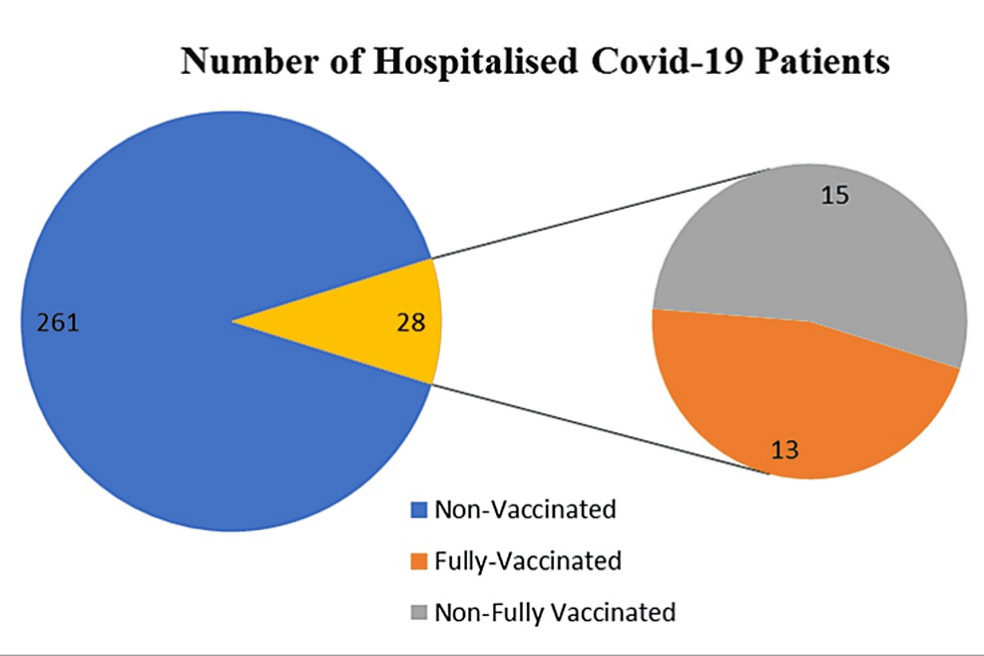
Vaccination status among hospitalized COVID-19 patients COVID-19: coronavirus disease 2019

Of note, 19.7% of the patients were intubated and an additional 29.1% were on high-flow oxygen (more than 15 liters/minute). The details of the hospitalized patients for COVID-19 pneumonia are presented in Figure 1; 90.3% of patients were non-vaccinated, 9.7% were partially vaccinated, and only 4.5% were fully vaccinated. Among the vaccinated patients, 82% had received the BNT162b2 vaccine, 14% had received the ChAdOx1 vaccine, and one patient had received the Chinese Sinopharm vaccine.

The mean age of fully and partially vaccinated patients was 65.36 (±14.29) years while the mean age of non-vaccinated patients was 52.65 (±17.84) years.

**Figure 2:**
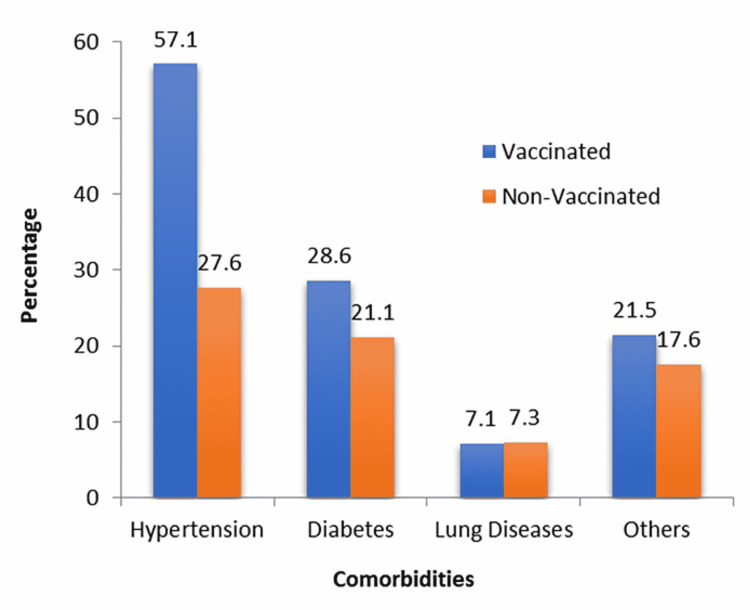
Comorbidities in hospitalized COVID-19 patients COVID-19: coronavirus disease 2019

Among the vaccinated group, a higher percentage of patients complained of chronic diseases compared to the non-vaccinated group, and the results are displayed in Figure 2. The p-value was significant for hypertension and non-significant for others (chi-squared test).

Regarding clinical findings, the mean duration from onset of symptoms to hospitalization was 7.76 (±3.6) days for non-vaccinated patients compared to 5.46 (±3.1) days for the vaccinated group. In addition, the length of stay among vaccinated patients was 16 days versus 12 days for non-vaccinated.

As for oxygen requirement, Table 1 shows the differences in oxygen needs between vaccinated and non-vaccinated patients.

**Table 1:** Oxygen requirements in hospitalized COVID-19 patients COVID-19: coronavirus disease 2019

			Vaccination status	
			Non-vaccinated	Vaccinated
Current need for O_2_	Intubation	Count	55	2
		% within vaccinated	21.1%	7.1%
	None	Count	6	2
		% within vaccinated	2.3%	7.1%
	O_2_ <15 liters	Count	124	16
		% within vaccinated	47.5%	57.1%
	O_2 _>15 liters	Count	76	8
		% within vaccinated	29.1%	28.6%

**Figure 3:**
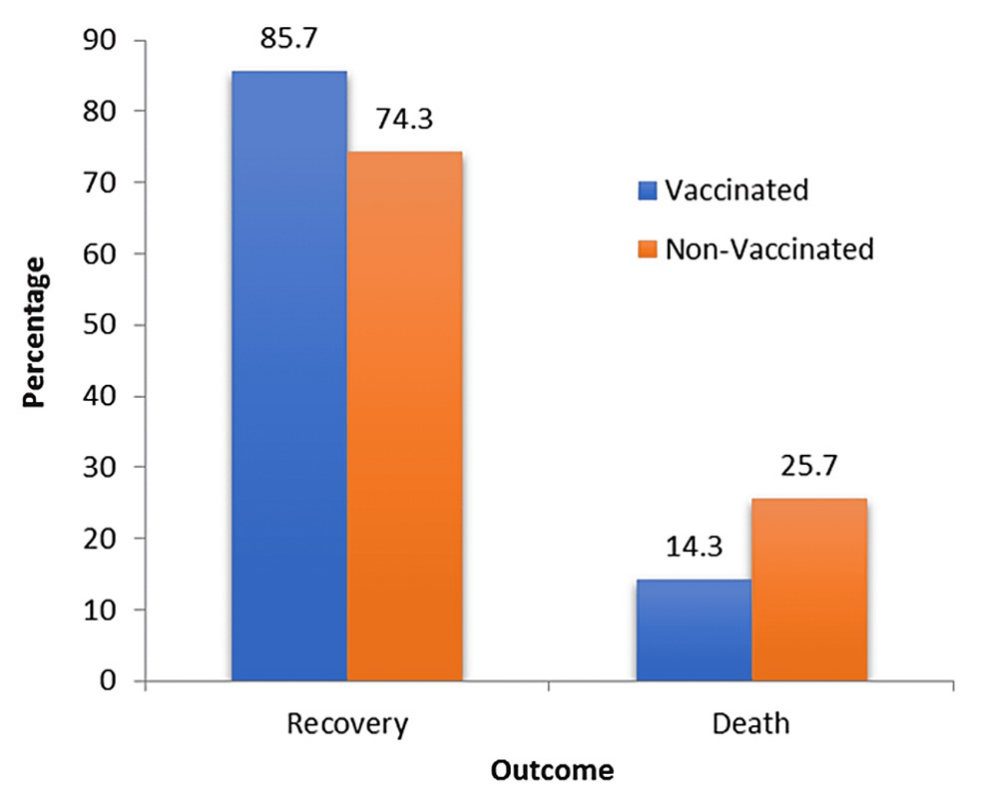
Outcomes among hospitalized COVID-19 patients COVID-19: coronavirus disease 2019

Concerning patient outcomes, the mortality rate was 24.6% of the total studied population; 25.7% of non-vaccinated patients died compared to 14.3% of the vaccinated patients (Figure 3). When pooling the non- and partially vaccinated patients, the rate of mortality was 25.1% compared to 11% among the fully vaccinated, and the difference was statistically not significant (p=0.742).

Table 2 shows the percentage of vaccinated population based on the daily reports of the Ministry of Public Health between July 1 and September 30, with a median rate of 22.5% for two doses in mid-August, and 27.6% for the first dose.

**Table 2:** The percentage of the vaccinated population

	First dose	Second dose
July 1, 2021	20.2%	10.2%
August 16, 2021	27.6%	22.5%
September 30, 2021	31.2%	25.8%

Therefore, the effectiveness of vaccines after the first and second dose was 71.71% and 83.78% respectively.

## Discussion

Ever since the declaration of COVID-19 as a global pandemic by WHO on March 11, 2020, the medical community initiated an intense search for cures and vaccines to combat the disease. Several vaccines associated with novel technologies were introduced for human trials and many have been approved for emergency use and have shown significant efficacy and acceptable safety profile. Many studies have been conducted to evaluate the effectiveness of the various COVID-19 vaccines after their distribution and use worldwide.

A typical vaccine is one that prevents the acquisition of the virus, its transmission, and progression to severe disease. The original data showed that the mRNA vaccine has an efficacy of more than 90% against symptomatic disease. Subsequent studies revealed that the immunity against the acquisition of the virus starts to decrease after around three months from the date of vaccination. Furthermore, new variants of COVID-19 have emerged, which are more capable of evading the vaccine [8]. In Lebanon, until the end of 2020, the ancestral strain of the virus was the most prevalent one. The Alpha variant took over in 2021 and Delta became the predominant strain from July 2021 until the beginning of 2022 when it was replaced by Omicron. During the period of this study, Delta was the dominant variant. The Pfizer/BioNTech BNT162b2 vaccine was the most commonly administered vaccine in Lebanon, followed by the Oxford-AstraZeneca ChAdOx1 nCoV-19 vaccine. A minority received the Russian Sputnik vaccine or the Chinese Sinopharm vaccine.

This study showed that full vaccination status had an effectiveness of 83.78% against hospitalization from COVID-19 during the Delta wave. This finding is consistent with that in the available literature. A study on the vaccine effectiveness among healthcare workers during the period of the predominance of the native virus strain showed vaccine effectiveness of 96% (95% CI: 82.2-99.1) against symptomatic infection seven or more days after the second dose [9]. Another study conducted in Belgium showed that when both an index case and contact were vaccinated, the vaccine effectiveness exceeded 90% [10]. A meta-analysis of large observational studies showed that the effectiveness of the bnt162b2 mRNA vaccine against symptomatic infection was 95% (95% CI: 96-97) more than seven days after the second dose [11].

When it comes to overall vaccine effectiveness, there was a considerable decline with the emergence of new variants of the virus. In a study from England, the effectiveness of two doses of the BNT162b2 vaccine was reported to be 93.7% (95% CI: 91.6-95.3%) among persons with the Alpha variant, which dropped to 88.0% (95% CI: 85.3-90.1) among those with the Delta variant. The effectiveness of two doses was even lower with the ChAdOx1 nCoV-19 vaccine. It was 74.5% (95% CI: 68.4-79.4) among persons with the Alpha variant and 67.0% (95% CI: 61.3 to 71.8) among those with the Delta variant [12]. In a cohort study of US frontline workers, the vaccine effectiveness reportedly dropped from 91% to 66% during the predominance of the Delta variant. Vaccine effectiveness against hospitalization remained higher but it dropped more than 120 days after the administration of the vaccine [13].

In fact, vaccine effectiveness does not decrease with the emergence of some variants that escape immunity but also remains potent three to four months after the vaccination. This correlates with dropping antibody titers with the increased risk of symptomatic infection and transmission. Protection from severe disease, probably related to cellular immunity, continues to confer protection for longer periods. However, this eventually decreases with the passage of time, especially in the elderly and the immunocompromised population who are unable to develop a robust immunological response [14].

In a prospective cohort study done by Hall et al. (the SIREN) in England, they calculated the duration of infection after being vaccinated and paired it with unvaccinated persons from December 2020 to February 2021. Effectiveness of 70% was observed after 21 days from the first dose of BNT162b2 compared to 85% after seven days of taking a second dose [15]. Similar results were shown by Bernal et al. who conducted a test-negative case-control study in an English community. Between December 2020 and February 2021, BNT162b2 and ChAdOx1 vaccine effectiveness were almost 60% after 28-34 days from the first dose, while the effectiveness of BNT162b2 rose to 89% 14 days after the second dose [16].

Pritchard et al. did a retrospective study in the UK to evaluate the effectiveness of the BNT162b2 and ChAdOx1 vaccines between December 2020 and May 2021. Their data demonstrated a 61% decrease in COVID-19 infection after 21 days of taking the first ChAdOx1 vaccine compared to 66% with BNT162b2. Taking the second dose led to a further reduction of 79% for ChAdOx1 and 80% for BNT162b2 [17].

As for the data collected in this study, we observed a higher rate of mortality in the unvaccinated population, with 25.7% deaths recorded among the unvaccinated patients vs. 14.3% among the vaccinated, and the most common cause of death was bacterial superinfections. The vaccinated group among hospitalized patients was older and had more comorbidities, mainly hypertension (57.2% vs. 27.6%) and diabetes (28.6% vs. 21.1%). The elderly, as well as patients with comorbidities, were the priority group for vaccination, and during that phase, hospitalization was restricted to severe cases unable to be cared for at home [18].

Vaccinated patients in this study had a longer length of hospital stay of 14.6 days compared to 12 days in those who were not. According to a study done in Norway by Whittaker et al., there was a shorter overall length of stay in the hospital for fully vaccinated patients and a lower risk of ICU admission. However, they failed to find a difference in length of stay in ICU or risk of in-hospital death between vaccinated and unvaccinated patients [19]. This result may be attributed to the higher mortality rate in non-immunized patients or the fact that the vaccinated elderly had more healthcare-associated complications. 

Regarding oxygen requirement in COVID-19 patients, an observational study done in Bangladesh on 174 vaccinated patients who were hospitalized for active COVID-19 showed that 58.2% of the fully vaccinated needed no oxygen compared to those who had received only one dose of AstraZeneca vaccine [20]. Based on this data, only two vaccinated patients required intubation compared to 55 among the non-vaccinated group (7% vs. 21.1% of the unvaccinated patients).

In summary, this study showed that the administration of vaccines in Lebanon during the Delta wave conferred robust protection against severe disease and hospitalization among the population.

Study limitations

It is essential to mention that the adopted screening method or case-population method is not the best design to be adopted for these types of studies. Moreover, our study was a small single-center study, limiting the ability to extrapolate the results to the general Lebanese population. Hence, future studies involving multiple centers with a larger data pool and using test-negative designs should be conducted to gain deeper insights into the topic.

## Conclusions

This retrospective study confirmed that vaccination against COVID-19 is highly effective in decreasing the risk of severe disease as manifested by hospitalization. Our findings showed that complete/partial vaccination in admitted patients reduced the length of hospitalization and the need for ICU admission either for increased oxygen therapy or ventilatory support at RHUH during the study period from July to September 2021. Our results align with other studies in the literature that endorse the effectiveness of SARS-CoV-2 vaccines when it comes to reducing the length of hospitalization and the severity of the infection.
